# Insufficient ER-stress response causes selective mouse cerebellar granule cell degeneration resembling that seen in congenital disorders of glycosylation

**DOI:** 10.1186/1756-6606-6-52

**Published:** 2013-12-04

**Authors:** Liangwu Sun, Yingjun Zhao, Kun Zhou, Hudson H Freeze, Yun-wu Zhang, Huaxi Xu

**Affiliations:** 1 Neurodegenerative Disease Research Program, Sanford-Burnham Medical Research Institute, La Jolla, CA, USA; 2 Fujian Provincial Key Laboratory of Neurodegenerative Disease and Aging Research and Institute of Neuroscience, College of Medicine, Xiamen University, Xiamen, Fujian, China; 3 Sanford Children’s Health Research Center, Sanford-Burnham Medical Research Institute, La Jolla, CA, USA

**Keywords:** Cerebellar granule cells, Congenital disorders of glycosylation, Cortical neurons, Endoplasmic reticulum stress, GRP78/BiP, Neurodegeneration, Phosphomannomutase 2

## Abstract

**Background:**

Congenital disorders of glycosylation (CDGs) are inherited diseases caused by glycosylation defects. Incorrectly glycosylated proteins induce protein misfolding and endoplasmic reticulum (ER) stress. The most common form of CDG, PMM2-CDG, is caused by deficiency in the cytosolic enzyme phosphomannomutase 2 (PMM2). Patients with PMM2-CDG exhibit a significantly reduced number of cerebellar Purkinje cells and granule cells. The molecular mechanism underlying the specific cerebellar neurodegeneration in PMM2-CDG, however, remains elusive.

**Results:**

Herein, we report that cerebellar granule cells (CGCs) are more sensitive to tunicamycin (TM)-induced inhibition of total N-glycan synthesis than cortical neurons (CNs). When glycan synthesis was inhibited to a comparable degree, CGCs exhibited more cell death than CNs. Furthermore, downregulation of PMM2 caused more CGCs to die than CNs. Importantly, we found that upon PMM2 downregulation or TM treatment, ER-stress response proteins were elevated less significantly in CGCs than in CNs, with the GRP78/BiP level showing the most significant difference. We further demonstrate that overexpression of GRP78/BiP rescues the death of CGCs resulting from either TM-treatment or PMM2 downregulation.

**Conclusions:**

Our results indicate that the selective susceptibility of cerebellar neurons to N-glycosylation defects is due to these neurons’ inefficient response to ER stress, providing important insight into the mechanisms of selective neurodegeneration observed in CDG patients.

## Background

Congenital disorders of glycosylation (CDGs) are inherited autosomal recessive disorders caused by defects in the glycosylation pathway, and display a broad spectrum of clinical features such as psychomotor retardation, hypotonia, intractable seizures, stroke-like episodes, internal strabismus, cyclic vomiting, hydrops fetalis, and failure to thrive [1,2]. There are about 70 reported gene defects that affect N-linked and/or O-linked glycosylation pathways, resulting in truncated or completely missing glycans and leading to the pathogenesis of CDGs. Mutations in the *PMM2* gene that encodes the cytosolic enzyme phosphomannomutase 2 (PMM2) result in the most common and well-known CDG, PMM2-CDG (or CDG-Ia), of which more than 800 cases have been reported worldwide. The physiological function of PMM2 is to convert mannose 6-phosphate to mannose 1-phosphate and a complete loss of PMM2 can cause lethality in yeast, mice and presumably humans. Cerebellar atrophy, or hypoplasia, is a major and nearly constant feature of PMM2-CDG [3-5]. Histological and immunocytochemical examination of cerebellar tissues from PMM2-CDG patients show partial atrophy of cerebellar folia with a severe loss of Purkinje cells and granule cells, and various morphological changes in the remaining Purkinje cells. However, it is unclear why cerebellar neurons are selectively susceptible to glycosylation defects in these patients.

Altered glycosylation in CDG leads to protein misfolding and induces stress in the endoplasmic reticulum (ER). ER stress is caused by an imbalance between the cellular demand for ER function and ER capacity [6-8]. Multiple physiological or pathological conditions that affect protein folding and/or calcium homeostasis can cause ER stress. These conditions include underglycosylation of glycoconjugates, glucose starvation, elevated protein synthesis and secretion, and failure of protein folding, transport or degradation. After sensing ER stress, cells activate the unfolded protein response (UPR) pathway to alleviate the problem and maintain function through two adaptive mechanisms: (i) increasing the folding capacity of the ER through upregulating the genes encoding molecular chaperones and foldases [9] and (ii) decreasing the protein burden on the ER through inhibition of protein synthesis and enhancing ER-associated degradation of misfolded proteins [10]. Thus, UPR enables the cells to reduce the misfolded protein load on the ER and promotes protein folding, secretion and degradation [6,11]. Genome-wide analysis of the UPR in fibroblasts from CDG patients show that CDG cells have chronic ER stress, and that the genes encoding components of the UPR are moderately induced [12].

Herein, we studied the molecular mechanism underlying the selective vulnerability of cerebellar neurons in PMM2-CDG. Our results revealed that murine cerebellar granule cells (CGCs) are much more sensitive to glycosylation defects and associated cell death than cortical neurons (CNs), and that a less efficient response to glycosylation disruption-induced ER stress in CGCs may be responsible for their selective vulnerability and neurodegeneration in the patients.

## Results

**Figure 1:**
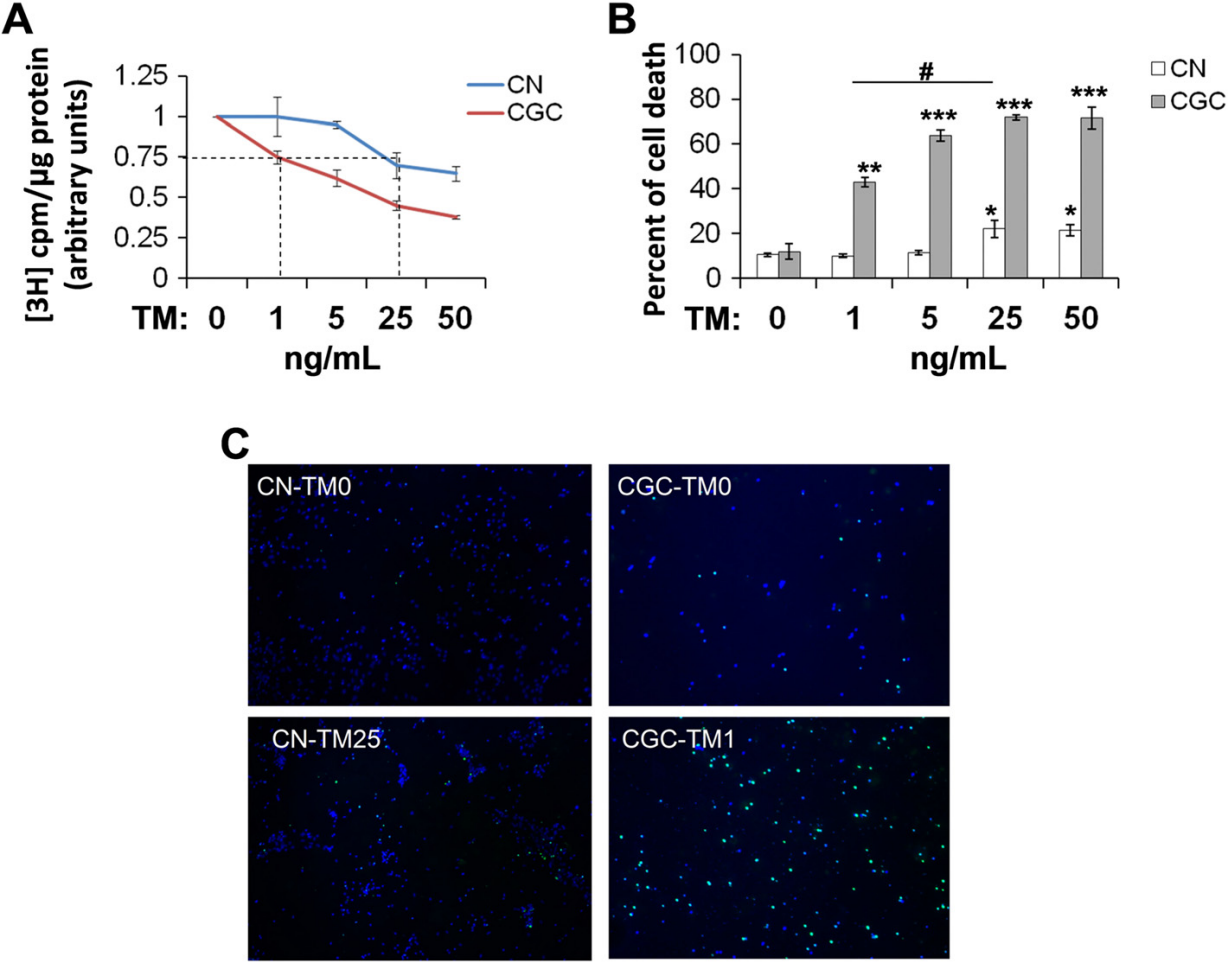
**CGCs are more sensitive to TM-mediated neurotoxicity than CNs. A**. Murine primary CGCs and CNs were exposed to various concentrations of TM for 3 d. Newly synthesized glycans were labeled for 20 h with [^3^H]-mannose and assayed, n = 3, error bars: standard deviation. Synthesis of glycans was decreased considerably more in CGCs than in CNs. Treatment with 1 ng/mL TM in CGCs resulted in a reduction in glycan synthesis (25%) comparable to that by treatment with 25 ng/mL of TM in CNs (indicated by dashed lines). **B**. CGCs and CNs were exposed to various concentrations of TM for 3 d. Cells were then subjected to a TUNEL assay to quantify cell death. TM treatments induced cell death in both CNs and CGCs. **: *p* < 0.01; ***: *p* < 0.001. When glycan synthesis was inhibited at a comparable level (25%), cell death in CGCs treated with 1 ng/mL TM was significantly more than that of CNs treated with 25 ng/mL TM. #: *p* < 0.01. **C**. Representative TUNEL staining of CNs and CGCs upon treatment with 25 and 1 ng/mL TM, respectively. Dying cells were stained in green and cell nuclei were stained in blue (with DAPI). TUNEL staining of cells without TM treatment (TM0) was used as a control. Please note that during cell culturing, the seeding densities for CNs and CGCs were different.

The nucleoside antibiotic tunicamycin (TM) is a specific inhibitor of N-linked glycosylation and has been widely used to study glycosylation defects [13,14]. Herein, we treated murine primary CGCs and CNs with various concentrations of TM and measured glycan synthesis in these neurons. The results showed that TM treatments inhibited glycan synthesis in both CGCs and CNs in a dose-dependent manner (Figure 1A). However, treatments with the same concentrations of TM resulted in a more dramatic reduction in glycan synthesis in CGCs than in CNs. Treatment with 1 ng/mL TM in CGCs led to a 25% reduction in glycan synthesis, whereas 25 ng/mL TM was required in CNs for the same level of reduction (Figure 1A). These results suggest that glycosylation in CGCs is more sensitive to TM than that in CNs.

We next studied the death of CGCs and CNs in response to TM treatments. Similar to glycan synthesis reduction, TM treatments induced cell death in CGCs and CNs in a dose-dependent manner (Figure 1B). However, low doses of TM (1–5 ng/mL) already induced significant death in CGCs, whereas only high doses of TM (25–50 ng/mL) caused significant death in CNs. Moreover, when glycan synthesis was inhibited to a comparable level (25%) (i.e., 1 ng/mL and 25 ng/mL TM for CGCs and CNs, respectively), cell death in CGCs was much greater than in CNs (Figure 1C) and the difference was statistically significant (Figure 1B).

**Figure 2:**
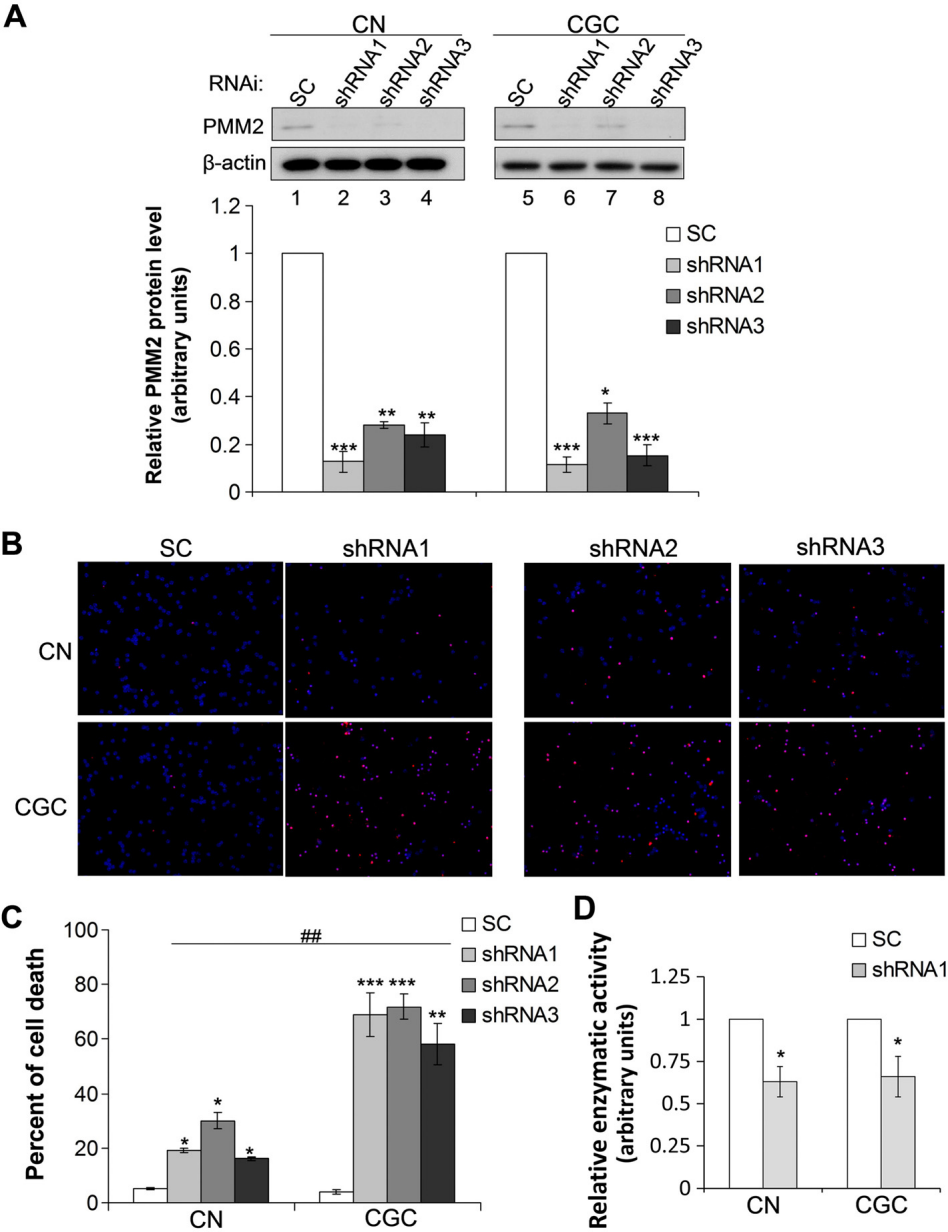
**Downregulation of PMM2 results in more cell death in CGCs than in CNs.** CGCs and CNs were infected with lentivirus containing PMM2 or Scrambled control (SC) shRNAs for 3 d. Cells were then subjected to Western blotting and densitometry to determine the protein level reduction of PMM2, n = 3, *: *p* < 0.05; **: *p* < 0.01; ***: *p* < 0.001 **(A)**; and TUNEL staining to study cell death **(B)**. Dying cells were stained in red and cell nuclei were stained in blue (with DAPI). **C**. After TUNEL staining, cell death percentages were quantified. Downregulation of PMM2 led to increased cell death in both CNs and CGCs, *: *p* < 0.05; **: *p* < 0.01; ***: *p* < 0.001. However, there was considerably more cell death in CGCs than in CNs. ##: *p* < 0.01. Please note that during cell culturing, the seeding densities for CNs and CGCs were different. **D**. CGCs and CNs were infected with lentivirus containing PMM2 shRNA1 or SC shRNA for 3 d, and then subjected to PMM enzymatic activity assay, n = 3, *: *p* < 0.05.

To explore the role of PMM2 in specific cerebellar neurodegeneration in CDG, we downregulated PMM2 expression using three different shRNAs in murine primary CGCs and CNs. When the levels of PMM2 protein were reduced in CNs and CGCs (Figure 2A), both types of cells underwent marked death (Figure 2B,C). However, the death rate in CGCs (~60-70%) was significantly higher than that in CNs (~20-30%) (Figure 2B,C), even when the protein level of PMM2 (using shRNA1, Figure 2A, lanes 2 vs 6) and the enzymatic activity of PMM (Figure 2D) were reduced to a similar extent in CNs and CGCs. These results suggest that CGCs are more vulnerable to neurodegeneration than CNs in response to PMM2 deficiency.

**Figure 3:**
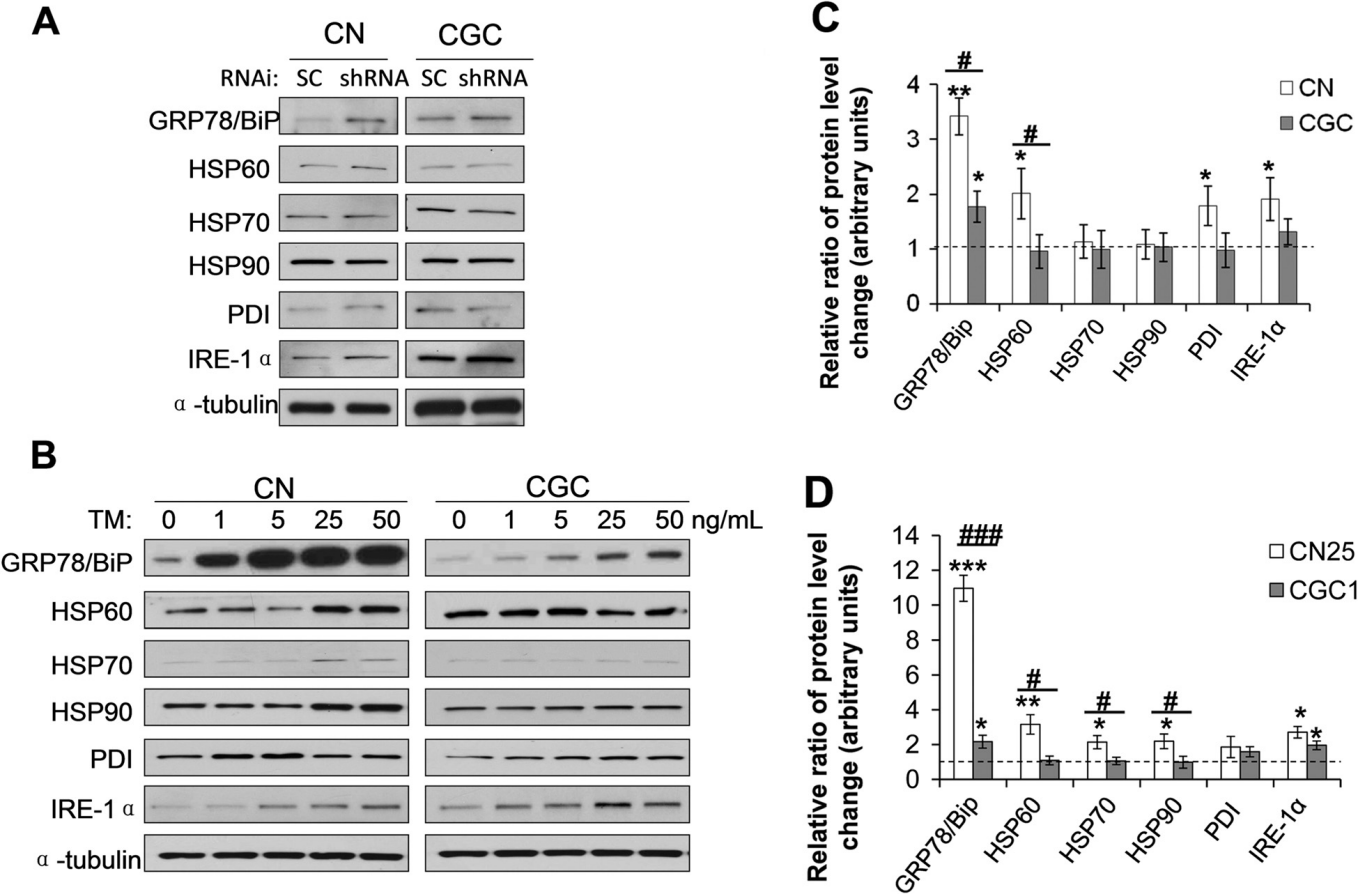
**CGCs and CNs show different ER stress responses to glycosylation disruption induced by PMM2 downregulation or TM treatments.** CGCs and CNs were infected with lentivirus containing PMM2 shRNA1 or scrambled control (SC) shRNA for 3 d **(A)**, or treated with TM for 3 d **(B)**. Equal amounts of cell lysate proteins were subjected to Western blotting with antibodies against different ER stress-response proteins. Protein levels were quantified by densitometry. **C**. Protein level changes upon PMM2 downregulation in *A* were determined in CNs and CGCs, respectively. **D**. Protein level changes upon treatment with 1 ng/mL TM in CGCs and upon 25 ng/mL TM in CNs in *B* were determined. The degree of protein level change within CGCs and CNs were compared to one (indicated by dashed lines), n = 3, *: *p* < 0.05; **: *p* < 0.01; ***: *p* < 0.001. The degree of protein level change between CGCs and CNs were also compared, n = 3, #: *p* < 0.05; ###: *p* < 0.001.

N-linked oligosaccharides are critical for protein folding [15,16]. It is well established that underglycosylation of glycoproteins causes protein misfolding and ER stress. Therefore, we studied the expression levels of ER stress-response proteins. We found that when the protein level of PMM2 (Figure 2A, lanes 2 vs 6) and the enzymatic activity of PMM (Figure 2D) were downregulated to a similar extent by shRNA1, the protein level of GRP78/BiP (glucose regulated protein/binding immunoglobulin protein) was increased in both CGCs and CNs (Figure 3A,C). However, the increase of GRP78/BiP level in CGCs (1.77 folds) was significantly lower than that in CNs (3.42 folds) (Figure 3C). Similarly, when cells were treated with TM, the protein level of GRP78/BiP was increased in both CGCs and CNs in a dose dependent manner (Figure 3B; Additional file 1: Figure S1). However, when glycan synthesis was inhibited to a comparable level (25%) (Figure 1A), the increase of GRP78/BiP level in CGCs treated with 1 ng/mL TM (2.17 folds) was significantly lower than that in CNs treated with 25 ng/mL TM (10.96 folds) (Figure 3D).

We also compared changes in the expression level of other ER stress-response proteins, including PDI, IRE-1α, and the heat shock proteins HSP60, HSP70, and HSP90, between CGCs and CNs. We found that when the level of PMM2 was downregulated in CNs, the expression levels of these proteins were increased to varying degrees. The increases in HSP60, PDI and IRE-1α showed statistical significance (Figure 3A,C). However, downregulation of PMM2 had no significant effect on these proteins in CGCs (Figure 3A,C). When CNs were treated with TM, we found that the levels of all these proteins were elevated in a dose-dependent manner, whereas only the levels of PDI and IRE-1α were increased in CGCs (Figure 3B; Additional file 1: Figure S1). In addition, the protein level increases in CGCs treated with 1 ng/mL TM were lower than those in CNs treated with 25 ng/mL TM, with the differences in HSP60 (1.09 folds vs. 3.16 folds), HSP70 (1.06 folds vs. 2.14 folds), and HSP90 (0.99 fold vs. 2.19 fold) showing statistical significance (Figure 3D). Together, these results suggest that CGCs have a less efficient response to ER stress induced by glycosylation disruption than CNs.

**Figure 4:**
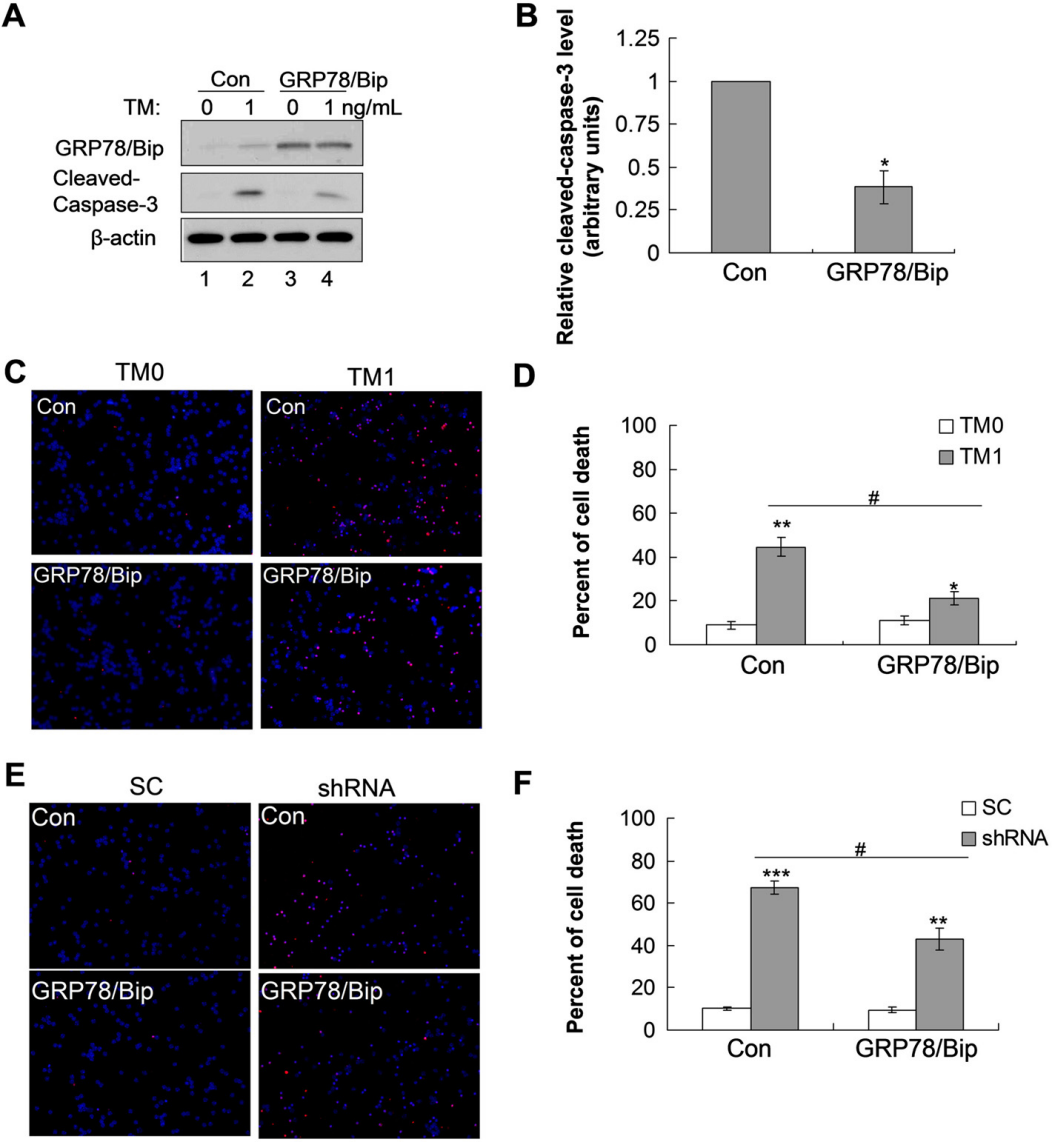
**Overexpression of GRP78/Bip rescues CGCs from cell death induced by TM treatment or PMM2 downregulation.** CGCs were infected with GRP78/Bip-overexpressing adenovirus or control (Con) adenovirus 1 d prior to TM treatment **(A-D)** or PMM2 shRNA downregulation **(E-F)**. **A**. Equal amounts of cell lysate proteins were subjected to Western blotting to determine the protein levels of GRP78/Bip and cleaved-caspase-3. **B**. Cleaved-caspase-3 levels were quantified by densitometry, n = 3, *: *p* < 0.05. **(C and E)** Representative TUNEL staining of CGCs upon TM treatment or PMM2 shRNA, respectively. Dying cells were stained in red and cell nuclei were stained in blue (with DAPI). **(D and F)** Quantification of cell death in **C** and **E**, respectively. *: *p* < 0.05, **: *p* < 0.01, ***: *p* < 0.001, comparison between TM- or PMM2 knockdown-treated and untreated samples. #: *p* < 0.05, comparison between GRP78/BiP-overexpressing and control samples.

Among the stress-response proteins studied, the difference in GRP78/BiP between CGCs and CNs, upon PMM2 downregulation and TM treatment to disrupt glycosylation, was the most striking (Figure 3C,D). GRP78/BiP is an essential ER chaperone that binds misfolded proteins to prevent them from forming aggregates and its expression is upregulated during ER stress [17]. Upregulation of GRP78/BiP expression is a key target of the UPR, and is required to alleviate ER stress and prevent ER stress-induced cell death. To further test our hypothesis that insufficient ER-stress response in CGCs is the underlying mechanism for the selective neurodegeneration of CGCs in PMM2-CDG, we carried out rescue experiments by modulating the levels of GRP78/BiP. Exogenous GRP78/BiP was overexpressed via an adeno viral expression system in CGCs, resulting in greater than 20-fold expression levels of exogenous over endogenous GRP78/BiP (Figure 4A, lanes 1 vs 3). Overexpression of GRP78/BiP significantly inhibited caspase-3 activation in CGCs treated with TM (~60%) (Figure 4A,B). Consistently, the death of CGCs induced by TM treatment was dramatically inhibited upon GRP78/BiP overexpression (Figure 4C,D). Likewise, cell death in CGCs subjected to PMM2 knockdown was significantly reduced upon GRP78/BiP overexpression (down from 70% to 42%, Figure 4E,F). Thus, our findings demonstrate that increasing GRP78/BiP levels in cerebellar neurons can largely, if not completely, inhibit cell death induced by ER-stress stimuli.

## Discussion

CDGs are a rapidly expanding group of inherited diseases with abnormal glycosylation. Patients with the most common CDG type, PMM2-CDG, develop cerebellar atrophy/hypoplasia, in which significant numbers of cerebellar Purkinje cells and granule cells are lost. Herein, for the first time to our knowledge, we provide a molecular mechanism to explain the selective cerebellar neurodegeneration in PMM2-CDG patients. We show that cerebellar neurons are selectively more vulnerable than cortical neurons to glycosylation defects triggered either by TM treatment or by PMM2 downregulation. The selective neuronal death of cerebellar neurons following glycosylation disruption seems to be due, at least in part, to insufficient induction of ER stress-response proteins, especially GRP78/Bip, a molecule that can prevent the aggregation of misfolded proteins during ER stress. Accordingly, we demonstrate that increasing GRP78/Bip levels in cerebellar neurons can largely prevent neuronal death caused by dysregulated glycosylation. Consistently, genetic disruption of the *SIL 1* gene that encodes a GRP78/BiP co-chaperone in woozy mutant mice leads to protein accumulation, ER stress, and selective cerebellar neuronal loss; homozygous woozy mutant mice develop ataxia between 3 and 4 months of age and have significant loss of cerebellar Purkinje cells [18]. Furthermore, cells expressing the disrupted yeast GRP78/BiP ortholog Kar2p lose the ability to respond to ER stress [19]. Therefore, our results indicate that GRP78/BiP may be a vital element in restoring ER homeostasis and cell survival in cerebellar neurons and a potential target for CDG treatment.

When glycosylation was disrupted by TM treatment and PMM2 downregulation, both percentage of cell death and GRP78/Bip expression were increased in CGCs and CNs. However, the increased rates in GRP78/Bip and other ER-stress response proteins were different between TM treatment and PMM2 downregulation. This is probably because TM blocks the synthesis of all N-linked glycoproteins [13,14] and thus induces strong ER stress, whereas PMM2 downregulation only interferes with the conversion of mannose-6-phosphate to mannose-1-phosphate that enters the N-glycosylation pathway and thus impairs selective glycosylation and causes a less intensive ER stress.

In addition to PMM2, there are other genes/proteins that also affect N-linked glycosylation. Phosphomannomutase 1 (PMM1) shares 65% homology to PMM2 and also converts mannose-6-phosphate to mannose-1-phosphate [20]. Interestingly, PMM1 is found to be predominantly present in brain, whereas PMM2 seems to be ubiquitously expressed [21]; and this raises a possibility that PMM1 is implicated in enhanced cell death and GRP78/Bip expression by glycosylation defects. However, PMM1 has not been found to be associated with CDG or any other disease. In addition, *Pmm1* knockout mice show no observable abnormal phenotypes as those found in *Pmm2* knockout mice [22,23]. These findings imply that PMM1 may not be essential for normal development. But whether PMM1 and other proteins mediating N-glycosylation contribute to enhanced cell death still deserves further investigation.

## Conclusions

CDG patients with PMM2 deficiency have specific cerebellar neuron loss. PMM2 converts mannose 6-phosphate to mannose 1-phosphate that enters the N-glycosylation pathway. Herein we demonstrate that cerebellar neurons are selectively susceptible to N-glycosylation defects and this is due to cerebellar neurons’ inefficient response to ER stress. Our results provide important insight into the mechanisms of selective neurodegeneration observed in PMM2-CDG patients.

## Methods

### Primary cell cultures

Murine CNs and CGCs were isolated from postnatal day 0 and 7–10 d old C57Bl/6 mice, respectively, and cultured as described previously [24,25]. All procedures were performed in accordance with the Guide for Care and Use of Laboratory Animals of the National Institutes of Health and were approved by the Institutional Animal Use and Care Committee of Sanford-Burnham Medical Research Institute.

### Analysis of [^3^H]mannose-labeled glycans

CGCs and CNs were exposed to various concentrations of TM for 3 d. Glycan synthesis was then studied as described previously [26]. Briefly, cells were labeled with 20 μCi/mL [^3^H]-mannose (Perkin Elmer) for 20 h in the presence of tunicamycin (TM). After washing with Phosphate Buffered Saline (PBS), cells were lysed by sonication and cellular proteins were precipitated with trichloroacetic acid. Incorporation of [^3^H]-mannose in glycans was measured by liquid scintillation. Three independent experiments were carried out and paired t-test was used for statistical comparison.

### TUNEL assay

Cells were fixed with 4% polyformaldehyde at room temperature for 20 min and permeabilized with 0.2% Triton X-100 in PBS for 5 min. Cell death was analyzed using the DeadEnd Fluorometric TUNEL System (Promega) or Click-iT TUNEL Alexa Fluor Imaging Assay (Invitrogen), following the manufacturer’s instructions. The numbers of total and dying cells were counted from five randomly selected regions and the percent of cell death was calculated for CNs and CGCs, respectively. Three independent experiments were carried out and two way factorial ANOVA test was used for statistical analyses.

### Pmm2 downregulation

Three short hairpin RNA (shRNA) fragments targeting murine *Pmm2* (5′-GCATACAAAGATGGGAAAC-3; 5′-GCAGATCTACGGAAAGAGT-3′; 5′-GGTGGCAATGACCATGAGA-3′) were cloned into the lentiviral vector pLentiLox3.7, which contains an EGFP reporter gene. A scrambled shRNA sequence that does not target any human or mouse genes was used as control. Lentiviruses were prepared according to the Lentiviral Expression System protocol (Invitrogen) and used to transduce CGCs and CNs.

### PMM activity

PMM activity was assayed using a previously described procedure [27]. The assay was modified by adding glucose-1,6-bisphosphate instead of mannose-1,6-bisphophate as a cofactor. Three independent experiments were carried out and paired t-test was used for statistical analysis.

### GRP78/Bip overexpression

GRP78/Bip-overexpressing adenovirus and control adenovirus were kindly provided by Dr. Randal Kaufman (Sanford-Burnham Medical Research Institute). In rescue experiments, CGCs were infected with the adenovirus one day before TM treatment or PMM2 downregulation.

### Western blotting

After various treatments, cells were lysed and equal amounts of cell lysate proteins were subjected to SDS-PAGE and immunoblotting. Antibodies used here included those against GRP78/BiP (Santa Cruz Biotechnology), IRE-1α (Cell Signaling Technology), PDI (Cell Signaling Technology), cleaved-caspase-3 (Cell Signaling Technology), heat shock proteins (HSP60, HSP70, and HSP90) (Assay Designs), α-tubulin (Sigma), β-actin (Sigma), and PMM2 (Novus). Protein levels were quantitated by densitometry. Three independent experiments were carried out. For statistical analyses, protein levels were normalized to those of controls (set as one arbitrary unit) and paired t-test was used for comparison.

## Abbreviations

CDGs: Congenital disorders of glycosylation; CGCs: Cerebellar granule cells; CNs: Cortical neurons; ER: Endoplasmic reticulum; PBS: Phosphate Buffered Saline; PMM2: Phosphomannomutase 2; PMM2-CDG: Phosphomannomutase 2-associated congenital disorder of glycosylation; shRNA: Short hairpin RNA; TM: Tunicamycin; UPR: Unfolded protein response.

## Competing interests

The authors declare that they have no competing interests.

## Authors’ contributions

LS, YZ and KZ carried out experiments. HHF, Y-wZ and HX designed the study. LS, Y-wZ and HX wrote the manuscript. All authors read and approved the final manuscript.

## Supplementary Material

Additional file 1: Figure S1 CGCs and CNs show different ER stress responses upon TM treatments. CGCs and CNs were treated with indicated amounts of TM for 3 d. Equal amounts of cell lysate proteins were subjected to Western blotting with antibodies against indicated ER stress-response proteins. Protein levels were quantified by densitometry and normalized to those treated with 0 ng/mL TM (set as one arbitrary units) for comparison. n = 3, error bars: standard deviation. Click here for file
