# Supplementary material for: Insufficient ER-stress response causes selective mouse cerebellar granule cell degeneration resembling that seen in congenital disorders of glycosylation
Source: Mol Brain. 2013 Dec 4;6:52. doi: 10.1186/1756-6606-6-52 (PMC3907076; doi:10.1186/1756-6606-6-52)
Supplement: Additional file 1: Figure S1 — CGCs and CNs show different ER stress responses upon TM treatments. CGCs and CNs were treated with indicated amounts of TM for 3 d. Equal amounts of cell lysate proteins were subjected to Western blotting with antibodies against indicated ER stress-response proteins. Protein levels were quantified by densitometry and normalized to those treated with 0 ng/mL TM (set as one arbitrary units) for comparison. n = 3, error bars: standard deviation. [file 1756-6606-6-52-S1.pdf]

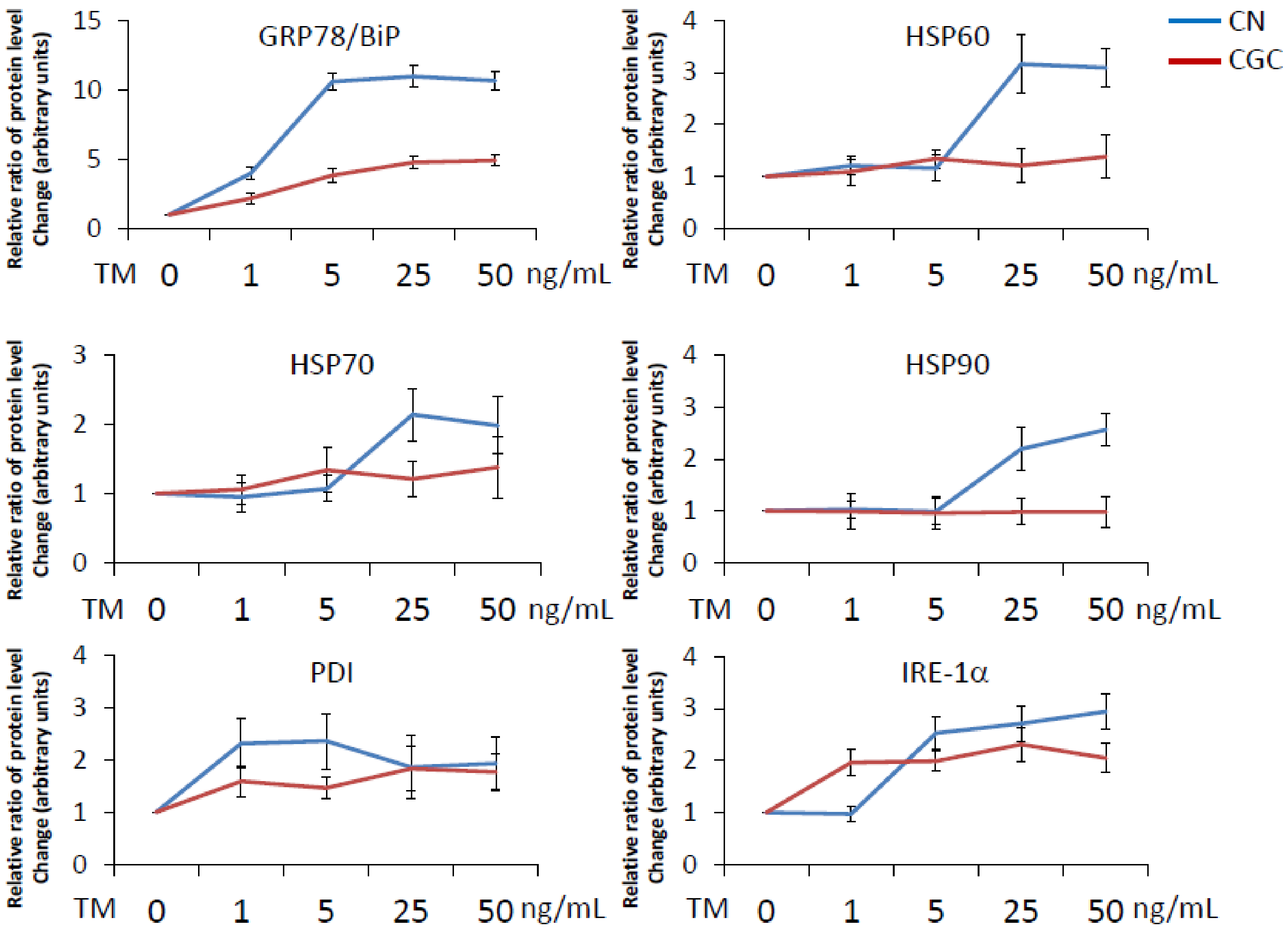

Figure S1 CGCs and CNs show different ER stress responses upon TM treatments. CGCs and CNs were treated with indicated amounts of TM for 3 d. Equal amounts of cell lysate proteins were subjected to Western blotting with antibodies against indicated ER stress-response proteins. Protein levels were quantified by densitometry and normalized to those treated with 0 ng/mL TM (set as one arbitrary units) for comparison. n=3, error bars: standard deviation.
